# Caution Is Required in Interpretation of Mutations in the Voltage Sensing Domain of Voltage Gated Channels as Evidence for Gating Mechanisms

**DOI:** 10.3390/ijms16011627

**Published:** 2015-01-12

**Authors:** Alisher M. Kariev, Michael E. Green

**Affiliations:** Department of Chemistry, City College of New York, 160 Convent Avenue, New York, NY 10031, USA; E-Mail: alisher@sci.ccny.cuny.edu

**Keywords:** voltage gating, quantum calculations, substituted cysteine accessibility method, proton transport, voltage sensing domain

## Abstract

The gating mechanism of voltage sensitive ion channels is generally considered to be the motion of the S4 transmembrane segment of the voltage sensing domains (VSD). The primary supporting evidence came from R→C mutations on the S4 transmembrane segment of the VSD, followed by reaction with a methanethiosulfonate (MTS) reagent. The cys side chain is –SH (reactive form –S^−^); the arginine side chain is much larger, leaving space big enough to accommodate the MTS sulfonate head group. The cavity created by the mutation has space for up to seven more water molecules than were present in wild type, which could be displaced irreversibly by the MTS reagent. Our quantum calculations show there is major reorientation of three aromatic residues that face into the cavity in response to proton displacement within the VSD. Two phenylalanines reorient sufficiently to shield/unshield the cysteine from the intracellular and extracellular ends, depending on the proton positions, and a tyrosine forms a hydrogen bond to the cysteine sulfur with its side chain –OH. These could produce the results of the experiments that have been interpreted as evidence for physical motion of the S4 segment, without physical motion of the S4 backbone. The computations strongly suggest that the interpretation of cysteine substitution reaction experiments be re-examined in the light of these considerations.

## 1. Introduction

Na^+^ and K^+^ voltage gated ion channels open in response to membrane depolarization. There is an accompanying gating current, a capacitative current preceding channel opening, typically about three charges per individual domain (about 13 charges for the four domains of the K^+^ *Shaker* channel, for example). Each voltage sensing domain (VSD) has four transmembrane (TM) segments. One, the S4 segment, has several arginines, spaced every third position on the segment, each pair separated by two hydrophobic residues. There are several “standard” models of the gating mechanism, having in common that the gating current consists of a move of the four S4 segments in an extracellular direction, sufficient for each to carry about three charges through the membrane field. A general review for the models as they stood over a decade ago contains most of the basic ideas in cartoon form [1]. While a huge amount of data and detail has been added since, the fundamental ideas concerning the mechanism of gating, and the source of gating current, remain similar. These models are, when taken as a group, considered essentially canonical, in spite of the fact that they disagree on many details of the S4 motion, as each fails to account for all the evidence. More recent models favor one or another of the versions; a huge literature has accumulated, to the point that it is no longer possible to refer to it comprehensively in a standard article.

We suggest that an alternate model, in which proton motion provides the gating current, is worth consideration. In order for this to be so, it is necessary that certain basic experimental work, in which cysteine is substituted for arginine, be reinterpreted. We present quantum calculations that give reasons for suggesting caution is in fact required in the interpretation of these experimental results. The calculations start from an open conformation, which allows more access of an external reagent to internal side chains, specifically of the cysteine, from the extracellular side of the membrane; an R→C mutation shows the appearance of a new cavity in the VSD as a result of the small size of the cysteine side chain compared to arginine. The consequences with several positions of protons in the cavity are calculated; the relevant protons are free in the calculation, but did not move. The results lead to the external reagent access without requiring motion of the S4 backbone. Absent backbone motion, there is no contribution to gating current, and the alternate possibility, H^+^ transport, becomes worthy of consideration. We are aware, of course, that other evidence exists that has been interpreted as supporting the standard model, as well as evidence that is easier to understand as proton motion than on the standard model, but reserve such discussion for another venue (but see the discussion of MD simulations below).

**Figure 1:**
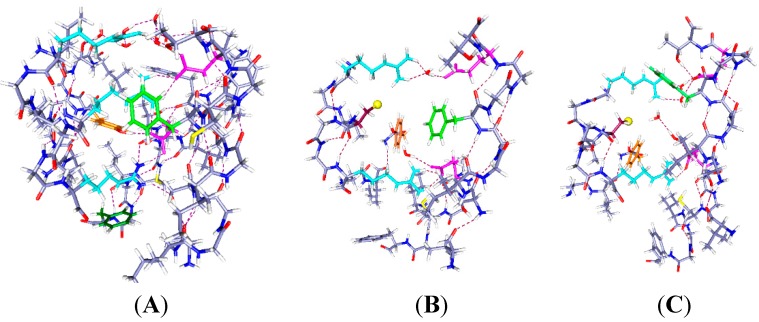
The central part of the voltage sensing domain, side view from R297 (**top**) to R303 (**bottom**) on the S4 TM segment, and nearby residues on S1, S2 and S3. Most of the protein is shown as dark blue (N) and gray (C). Arginines 297 and 303 are light blue, tyrosine (Y266) is orange, phenylalanine F233 green; in (**B**,**C**), C300 has the sulfur as a large yellow sphere. Glutamate side chains are magenta. The water molecules are red (oxygen) and white (hydrogen). Hydrogen bonds are dashed lines. A preliminary version of this figure was posted on arXiv [38]). Parts (**B**,**C**) are optimized with HF/6-31G** from a starting position that allowed salt bridges to be maintained where possible; part (**A**) was similarly optimized directly from the 3Lut structure, and the changes are small.

There has been experimental work reported that is directly relevant to those amino acids in the VSD with aromatic side chains, which are the ones that prove especially important in our results. The most work has appeared on the F233 residue of the K_v_1.2/2.1 chimera (F290 of *Shaker*) [2,3,4]. The most important experimental evidence for the standard models comes from experiments in which an arginine is replaced with a cysteine. The cysteine can ionize, leaving an ion with a side chain consisting of just –S^−^; unlike the neutral –SH species, the S^−^ can react with MTS reagents (un-ionized SH is about nine orders of magnitude slower, as the MTS reactive group is electrophilic) [5]. The R→C mutation leaves a cavity with a size essentially the difference between the single atom cysteine side chain and the very large guanidinium side chain of arginine. The cavity suffices to hold several water molecules, which in turn may be displaced if a reagent—such as MTS that can react irreversibly with the cysteine—is introduced. The MTS reagents may have various groups, positive, neutral, or negative, attached to the thiosulfonate head group. The method, originally used to study the lactose lacY transporter by Kaback and coworkers (van Iwaarden* et al.* [6]), was applied to ion channels (albeit ligand gated, not voltage gated, channels) by Akabas *et al*., who labeled it the substituted cysteine accessibility method (SCAM) [7]. They made three explicit assumptions: (1) The cysteine residue is either at the water accessible membrane surface, the lipid accessible surface, or the protein interior; (2) Hydrophilic reagents react faster at the water accessible surface; (3) Because the reaction with electrophiles like MTS reagents is so much faster with the ionized form than with the un-ionized form, and ionization is much more probable in a high dielectric medium, reaction only occurs at one of the water accessible membrane surfaces. They did not consider the possibility that an R→C, or other substitution of cysteine for a large side chain, would produce a cavity of up to roughly 200 Å^3^ (see Figure 1 in results), large enough for two, up to possibly seven, water molecules to enter, very possibly invalidating the third assumption. It is this point that we address here. In the original application by Karlin, Akabas, and coworkers, the amino acids for which the substitution was made were not as large as arginine, the largest being threonine, so that this issue was less significant. Horn and coworkers applied the method to voltage gated channels [8], and it has been used by multiple groups since. With voltage gated channels, the principal substitution has been for the S4 arginines, which, in the standard models of gating, are presumed to carry the gating charges as they move across the electric field of the membrane. Horn recognized that the third assumption might not be valid, but his interpretation of the results essentially requires that assumption; if the assumption is not made, the modeling of the S4 motion no longer holds. If the reaction need not be at the membrane surface, the S4 need not move during gating. So far as we are aware, none of the literature has considered the consequences of the discrepancy in size of the cysteine and arginine side chains. Not all cysteine substitution mutations leave a cavity. For example, substitution of cysteine for alanine or glycine would leave no cavity, but none of the definitive experiments involve these; as we noted, some substitutions leave only a small cavity, as in the original work of Akabas* et al*. [7]*.* Some experiments from the MacKinnon laboratory [9,10] were not limited to arginine substitutions, but their primary evidence still requires similar cautions. We will consider a specific R→C mutation, in the pdb 2A79 and 3Lut structures [11,12]. This will allow us to evaluate the consequences of an R→C mutation for the availability of the cysteine side chain to MTS reagents. A second consequence of a cysteine substitution is that salt bridges, as well as hydrogen bond networks, could be disrupted. This is very likely with an R→C mutation in the VSD, as the arginines are salt bridged to aspartates and glutamates on the other transmembrane segments, with the wild type guanidinium side chain stretching across the space that becomes the cavity in the mutant. Other amino acid substitutions that alter the arrangement of water molecules would disrupt hydrogen bonds, with uncertain consequences; other cysteine mutations may also be difficult to interpret, but we do not consider these mutations here. Several of the standard (S4 moves up) models of gating require the S4 arginines to exchange partners with the acidic residues on S2 and S3; with these salt bridges disrupted by substitutions, it introduces a further difficulty in understanding the applicability of the mutant results to the models.

We have suggested in previous work that proton motion is the source of gating current [13,14,15,16,17]. This carries the implication that the local field within the VSD moves with the protons, the section near the protons becoming locally positive. One consequence is that the direction of the field experienced by the substituted cysteine in the VSD is the reverse of the external field, so that its negative charge should alter the orientation of the C–S^−^ bond; however, other effects are considerably more important, especially the rearrangements of aromatic groups in the cavity. We here show the results of quantum calculations in the cavity left by the R300C mutation in S4 of a VSD. Several molecules of water, and enough of the protein to define the cavity, are present. The role of the protons is tested with 0, 1, or 2 protons, for several cases. The number of protons per domain should be about three, as the total gating current is approximately 13, added over four domains. A third proton would be likely to be distant enough that it would not be in the limited region that can be calculated; specifically there is a hydrophobic section near F233, within the VSD, and the third proton would be expected to be on the intracellular side of this section, even in the open state, while R300 is on the extracellular side. It is possible for protons to go through this section, as an R→H mutation at the end of the segment allows a proton current completely through the VSD [18]. Although the major contribution to the field is likely to be from the near protons, the third proton may have significant effects in raising the potential beyond a minimum required for other proton transfers, altering their direction, or rearranging the hydrogen bonds, with consequences that stretch into the region being calculated, even if the proton is two water molecules distant. To actually calculate these effects would require extensions of the system that are beyond the possibility of computation at the present time. We expect the neglected effects to be smaller than the large effects of the less distant protons. The calculation shows which residues are most affected by proton position; rotations of the aromatic residues in particular suggest major consequences for the ability of the cysteine side chain to react, not because C–S^−^ bond orientation changes with respect to the rest of the protein, but because rotations of other side chains may block or unblock the sulfur. The aromatics, a phenylalanine, F180, from the S1 TM segment plus another phenylalanine, F233, and, in some cases a tyrosine, Y266, similarly rotate in response to proton positions. In addition they rotate differently depending on the number of water molecules present, if the protons are omitted; the absence of protons may not be realistic, but it is useful to understand how sensitive the system is, in the absence of the anchoring guanidinium side chain. The two phenylalanines and the tyrosine are not the only VSD aromatic residues, but these are the ones that point into the cavity. If the external reagent can displace the water, which seems probable, the dependence of the position of the phenylalanine and tyrosine rings on the positions of the H^+^ ions would account for state dependent access of the external reagents in the cysteine substitution experiments. Water in the selectivity filter of the channel, and by extension the pore cavity, has recently been shown to have a very long lifetime [19,20], on the order of seconds. No corresponding work on water in the VSD has been reported, to the best of our knowledge, and the lifetime to be expected for at least some of the water would be the transit times for ions, of the order of 10^−7^ s. However, it would be unsurprising if the VSD had fairly long lived water as well, so that the position of water, as well as of protons, is important to gating in the WT (wild type) as well as the mutant channel. The mutant channel might have water at the protein surface with lifetimes in excess of 10^−7^ s. There is also evidence from neutron scattering on nanoconfined water (held in spaces under 2 nm) that protons have wave functions that differ from those in bulk, being more spread out [21]; the cavity left by the R→C mutation is approximately1 nm in its largest dimension. (We note that the first paper, to the best of our knowledge, to propose long lived water as a key part of the activation gate of the channel was published in 1989 [22]).

The state of water is clearly important, and affects another of the main lines of research that attempt to work out the gating mechanism, that is, simulations. For the most part these are molecular dynamics (MD) simulations [2,23,24,25,26,27], with others using Rosetta, or coarse-grained, or other forms of modeling [28,29,30,31,32]. Several of these point to 3_10_ helices as part of the gating mechanism, others do not. The modeling, including MD, uses several potential (*i.e*., force field) models, and these too may have problems [33]. At least one simulation finds water controls one aspect of channel state transitions, slow inactivation [20]. The extent of movement of S4 that these simulations find varies. A recent paper by Li* et al.* [25], suggests a relatively small motion, of the order of one helical “click”, that is, the S4 segment moves up one helical turn. Another recent paper [34] suggests a much larger displacement, based on one-dimensional electron density measurements, supplemented by MD simulation. These papers appear to be mutually contradictory, although, as they apply to different channels, it might be argued that different VSDs operate through significantly different mechanisms. They each offer evidence that would contradict our model as well, not based on R→C mutation, but each with a separate set of assumptions. We believe that there are alternate interpretations of each of these, but reserve discussion of them for future work. No simulation results or quantum calculations as yet, as far as we are aware, apply to R→C S4 mutants. Here, we present calculations that specifically consider the mutants, and show that these mutants could behave in such a way as to provide the experimental results, without the protein backbone moving.

Our calculations give the reason for requiring caution in interpreting the effects of an R→C mutation, and we suggest a possible alternative interpretation.

### Computational Details

All optimizations were done using HF/6-31G**, with either Gaussian [35] or NWChem [36]; some calculations were done partly using one, partly the other. The cases with 0 H^+^ had 386 atoms (2 water molecules) or 392 (4 water molecules), with 3848 and 3898 basis functions, respectively. A larger system was used for the cases with 1 or 2 H^+^: 1 H^+^, 489 atoms, 4823 basis functions; 2 H^+^ 493 atoms, 4853 basis functions. Charges on the entire system were: *q* = −1 + *n* (protons). Single point calculations to get energy were done on the optimized structures using B3LYP/6-311G**.

## 2. Results and Discussion

The main results on structure are given in the figures and Table 1 and Table 2. Table 3, with energies of the several configurations, helps also to understand the states that can be reached by the VSD, hence offer a check on whether the model is reasonable from the standpoint of the energy needed for transitions between pairs of states. The region from R297 to R303 of S4 (pdb: 3Lut) is shown in Figure 1, together with corresponding sections of the other TM segments of the same VSD; side chains that point away from the cavity, including several aromatic residues, are omitted from the calculation.

### 2.1. Results with no Protons in the Cavity

In Figure 1A, Wild type: Because the central arginine stretches across the space between S4 and the rest of the VSD, it appears that an external reagent would have difficulty entering the space. There could be very few water molecules in the center of the region, if any. It appears that the only place for water molecules is the top salt bridge, which is fairly weak, with approximate distances 5 to 6 Å. Based on earlier calculations on salt bridges [37], the protons are not completely transferred from guanidinium to acid. To some extent they are shared, making the effective bond stronger than in a side chain which has access to water, and fully ionizes. In the absence of a mutation, the S4 would have to move to allow an arginine to reach the surface to react; it could not react in its original position, making the interpretation unambiguous. However, experiments on accessibility have not been done on WT.

In Figure 1B, the R300C mutation, with two water molecules: The water molecules both form hydrogen bonds, one with R297 and its salt bridged glutamate (E183), with the oxygen of the water equally hydrogen bonded to two hydrogens of the arginine; the other water is near R303. The sulfur of the cysteine of the R300C mutation is doubly hydrogen bonded, to Y266 and to R297, and held to the side of the cavity; the distance of S to the nearest R297 atom is 3.56 Å, suggesting a fairly strong bond, essentially a salt bridge with cys as the acid, and the proton transferred. The F180 ring rotates into the cavity, largely filling it. Y266 is in the space below the cysteine. F233 at the bottom of the figure has also reoriented. This conformation leaves space for more water molecules.

**Figure 2:**
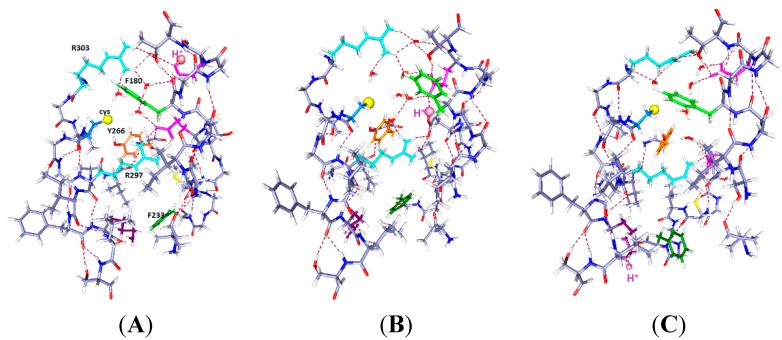
Three cases with one proton and six water molecules each: The colors are as in Figure 1. The H^+^ position is indicated by a large pale orange sphere, as well as being labeled H^+^, and the water molecules are red and white. Proton positions are: (**A**) H^+^ near the extracellular end (on E183); (**B**) H^+^ near mid cavity, on E226; The side chains of at least two key aromatics have moved significantly compared to where they were in Figure 1A. F233 has moved to a position distant from K306; and (**C**) H^+^ near the intracellular end, on K306. The reverse of the relevant positioning found in Figure 2A and B is true. The R297 side chain has rotated, but not translated, with the H^+^ down (**C**). The accessibility of the cysteine residue to an external reagent is different, possibly reversed completely, in the two cases. Overall, one can see that the major shifts are the aromatic residues, F233 and F180, while the arginine side chains hardly move. As in Figure 1, the view is from the side, with R297 at the top. Optimization: HF/6-31G**.

**Figure 3:**
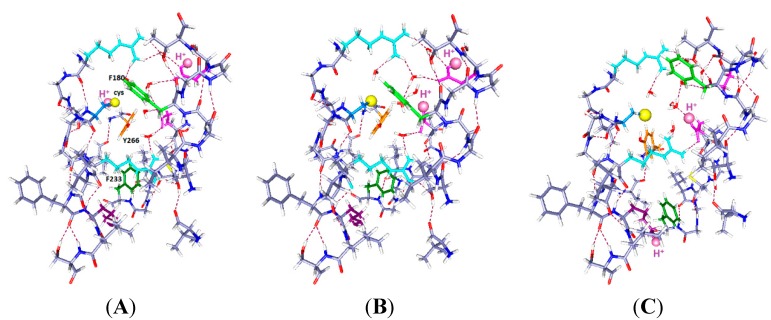
The same system with two protons and one more water (total 7 water molecules), with the same color scheme as in Figure 1 and Figure 2, and corresponding labels: (**A**) H^+^ on C300 and E183; (**B**) H^+^ on E183 (in S1) and E226 (in S2) (up and middle); and (**C**) H^+^ on E226 and K306 (down and middle). Note the change in positions of the F180 and F233 side chains as well as the rotation of Y266 in going from (**B**) to (**C**).

In Figure 1C, the R300C mutation with four water molecules: There are two water molecules now bridging R297 to E183, and two in the cavity center, one of them hydrogen bonded to the cysteine sulfur and to the other water molecule, which stretches across to the glutamate (E226) on the other side of the cavity. The sulfur atom now points into the cavity, with its second hydrogen bond to tyrosine (Y266), the side chain of which has itself moved appreciably again. The cysteine appears to be available to a reagent in the cavity. The F180 ring has folded sharply out of the way, as shown by the dihedral angle defined by atoms ring C4, ring C1 (the link to the atoms toward the backbone) and the next two atoms of the side chain toward the backbone, which is +171.3°, compared to −99.1° in the two water case. The Y266 ring has moved somewhat back from where it was in Figure 1B. The two additional water molecules make a major difference in conformation of the side chains. F233 shows more limited motion in the 0 H^+^ case than it does with H^+^ present (Figure 2 and Figure 3), and is not specifically labeled in Figure 1.

In the wild type channel, the arginine fills the space that will become the cavity in the mutant; the arginine is salt bridged and hydrogen bonded, which makes it fairly secure, as should be expected if the VSD is well structured, but not if the VSD is highly mobile. There does not appear to be a loose end in the region, especially for the arginine. Substantial activation energy would be needed to move this residue with respect to the groups to which it is hydrogen bonded and salt bridged, as breaking each of these would cost about 15–17 kJ·mol^−1^, assuming normal strength bonds on average (this is not a simple question, as the water affects both hydrogen bonds and salt bridges, but there is little reason to believe these bonds should be unusually weak, on average; when one bond is weaker, or longer, another must be shorter, hence stronger). In the mutant, where the cysteine residue does not come close to filling the space, rearrangements are much easier, even if translation to the surface is still difficult for S4, probably too difficult to consider. In Figure 1B,C, we can already see that the presence of four water molecules is enough to cause the rotation of the side chain of a phenylalanine, F180 of segment S1, out of the cavity. With only two water molecules, the F180 side chain can point into the cavity in the mutant. The four water molecules in the space would have to be displaced by the reactive end of an MTS molecule, to allow reaction with the cysteine. In the two water molecules case, the F180 side chain blocks the MTS reagent, although it could presumably be pushed out of the way. The positions of the aromatic rings are dependent on the number of water molecules as well as the position of protons, as we shall see. In addition, the side chains of Y266 and F233 are also mobile, and rearrange, bending through a large angle in the mutant. The hydrogen bonding to the reactive cysteine residue changes as the tyrosine moves. In the wild type, the two side chains stay well out of the space occupied by the guanidinium of R300. With two waters in the R300C mutant, both side chains move in to partially fill the cavity left by the mutation, and with four waters, the side chains move partially back, more of the space being filled by water, which can presumably be displaced. There is actually room for still more water in the cavity; we have calculated up to six waters with one proton, and, by adding an H_3_O^+^ to include a second proton, seven waters for two protons.

### 2.2. One Proton in the Cavity

In Figure 2, we see the results of placing a single proton in the cavity, in three different positions (we will use “up” for proton position to correspond to positions near the extracellular end, and “down” to correspond to the intracellular end): up (on E183), middle (on E226), and down (on K306). With the protons in the up and intermediate positions, the Y266 ring fits neatly under the sulfur; in the down case, the ring rotates, while keeping the –OH–S hydrogen bond distance effectively unchanged. The cysteine C–S bond has rotated only slightly toward the charge in going from Figure 2A to Figure 2B,C, a relatively small change by comparison with the aromatic ring motion. Most important, with the H^+^ intracellular, F180 forms a lid over the cavity, blocking it at acids that rotate or take an H^+^. These amino acids are in corresponding positions in Figure 2B,C. Note the positions of F180 in the three cases: It moves in or out of the cavity in response to proton position. With the single proton up (Figure 2A) on E183, the F180 folds back, and the Y266 rotates to an extended position on the S1 side of the cavity; this represents an almost 90° rotation with respect to the WT, a major shift in orientation. What seems to change at most minimally is the distance of the hydroxyl oxygen to the cys sulfur. The tyrosine is a weaker acid than cysteine (side chain p*K* 10.5, compared to 8.4 for cysteine [39]). If an H^+^ is to be lost, the cysteine will lose it, hence be able to react. Whether the tyrosine-cysteine combination forms a slightly stronger acid, allowing cysteine to react more easily, is not certain. F233 blocks the cavity intracellularly. With the proton part way up (Figure 2B), the F180 is at least as far out of the way, but the Y266 has rotated back so that the ring is closer to the sulfur. The distance of the F233 from a lysine on S4, K306, changes drastically. Finally, with the H^+^ down, which should correspond to channel closed (Figure 2C), the tyrosine ring is directly under the sulfur and the F180 phenylalanine has moved to fill the cavity, blocking access from the extracellular side. The F233 side chain also rotates to follow the proton, leaving space on the intracellular side. In other words, this time we are able to see a difference depending on the position of a proton. Therefore the dependence of the availability of the cysteine sulfur for reaction with an MTS reagent can be associated with the transfer of a proton. The Y266 ring rotates so as to cover the cysteine from the bottom when the proton is down, as it would be in the closed state, while maintaining the –OH–S hydrogen bond; this is consistent with the cysteine being unavailable from the top when the channel is closed. The rotations are consistent with the hypothesis that the availability of the cys depends on ring orientation, and shows that a model in which the S4 backbone moves physically is not only improbable because of the energetic cost, but unnecessary. With another proton, the changes are somewhat different, as shown in the next section.

### 2.3. Two Protons in the Cavity

Figure 3 shows the calculation with two protons plus a seventh water, as H_3_O^+^ is added. If two protons transfer when the membrane depolarizes, this would account even more easily for the observed state dependence of the cysteine reaction, maintaining observed gating current, and not requiring S4 motion.

The results in Figure 3 show somewhat larger motion of some side chains than in Figure 2. Only the key aromatics, plus the mutated cys and H, are labeled in Figure 3A, and the H is labeled in all three panels. Optimization is again HF/6-31G**. The R303 side chain has shifted slightly upward and shows some rotation, but the backbone has not moved. In Figure 3A, with neutral cysteine: There could be no reaction with an external reagent, as the negative ion of cysteine reacts, not the neutral species, which is included in this part of the calculation. Figure 3B, with one H^+^ up, one middle: F233 follows the charge, and F180 moves back from the cysteine. There is space above the cysteine, so an MTS reagent may be able to react with the cysteine if it comes from the extracellular side. The intracellular path appears blocked. Figure 3C, with one H^+^ down, one middle: F180 moves back, somewhat as in Figure 2C, but with a larger motion here, leaving space below the cysteine. It is not clear that there would be a path from the intracellular side, but now it appears at least possible. The Y266 hydroxyl remains hydrogen bonded to the cys S^−^, but the ring rotates, so it is not clear that any reaction is possible.

Below, the phenylalanine F233 rotates upward toward the protons. As in Figure 2, F233 rotates essentially out of the cavity when the proton(s) are down. With protons down as in Figure 3C, the rotation of the aromatic ring leaves space that might fit a reagent in from below, something that appears to be impossible in either of the other two positions. While a “middle” (E226) proton may correspond to an open or a closed state, it is useful to know how the rings respond to possible intermediate states that could correspond to pre-open states; such states are required by kinetic models of gating. E226 is also a plausible residue to hold a proton.

The calculation covers several possible positions for a proton, and then for a pair of protons. Places that protons could occupy are clear, and the water hydrogen bonds show some of the possible paths along which transfer of protons is possible. As we noted earlier, we know that protons can move within the VSD, from experiments in which an end arginine is mutated to histidine, allowing a proton current completely through the VSD [18,40], we know that proton transport within the VSD is possible. This is reinforced by proton transmission of the H_v_1 channel, which strongly resembles a VSD [41,42,43]. The assumption of proton movement therefore has experimental support.

The qualitative statements based on the figures can be understood more clearly by considering the changes in certain interatomic distances that show how the residues become closer or more distant with shifts in position of the protons. Table 1 and Table 2 show some of these distances. Another distance of interest, the Y266 side chain oxygen to cys S, stayed in a narrow range from 3.22 to 3.36 Å, except in the one case in which the H was on the S, and there was one other H^+^ on E183, in which case it expanded to 3.53 Å. In short, in spite of the rotations of the tyrosine ring, the oxygen-sulfur distance remained essentially fixed; the S to NH1 of R297 also hardly changed (Table 1 and Table 2). Therefore the cys S was one of the least mobile atoms in the structure, suggesting that what affected the reactivity of the sulfur was the rearrangement of the groups that covered it, especially the two phenyl groups, and their effect on the water.

**Table 1:** Certain distances (Å) between key atoms, 1 H^+^ case. Interatomic distances in which the position of the proton makes a difference of at least 3 Å are italicized. All such cases involve F180 or F233.

1 H^+^ (Location Shown)	H^+^: Up (E183)	H^+^: Middle (E226)	H^+^: Down (K306)
*C_A_ (R297)–C_Z_ (F180)*	*7.85*	*10.82*	*5.72*
NH1 (R297)–S (C300)	5.75	5.52	5.52
NH1 (R297)–C_Z_ (F180)	4.84	7.59	6.21
OE2 (E183)–C_Z_ (F180)	5.74	7.25	5.29
*N (R303)–C_Z_ (F233)*	*11.14*	*6.65*	*6.73*
*N_Z_ (K306)–C_Z_ (F233)*	*3.84*	*6.85*	*6.85*
N (R303)–K (306)	10.94	11.49	10.82

**Table 2:** Certain distances (Å) between key atoms, 2 H^+^case. Interatomic distances in which the position of the proton makes a difference of at least 3 Å are italicized. All such cases involve F180 or F233.

2 H^+^ (Locations Shown)	H^+^: Up + Mid (E183, E226)	H^+^: Mid + Down (E226, K306)	H^+^ (Up) E183 + Cys300
C_A_ (R297)–C_Z_ (F180)	7.02	8.83	7.23
NH1 (R297)–S (C300)	5.43	5.94	5.42
NH1 (R297)–C_Z_ (F180)	5.32	6.18	5.58
OE2 (E183)–C_Z_ (F180)	6.17	5.92	6.04
*N (R303)*–*C_Z_ (F233)*	*3.65*	*6.87*	*4.20*
*N_Z_ (K306)*–*C_Z_ (F233)*	*7.34*	*4.04*	*7.35*
N (R303)–K (306)	9.40	10.20	8.99

Phenyl groups, hence phenylalanine side chains, rotate toward a positively charged group; the ring π-electrons will complex with cations, more strongly with the cation centered above the ring, although they also may complex edge on; the orientation matters, but the π-electrons are still able to produce an attraction. The π-electron attraction has an interesting possible corollary: It is known [4] that the F233 is involved in gating, based on several mutations. The one that left-shifts gating, making the channel open with less depolarization, is F233W, in which the single phenylalanine ring is replaced with the two rings of the tryptophan side chain (which also has a nitrogen, making it a weak base). This presumably makes the orientation even more sensitive to displacement of charge within the VSD. The rotation of F233 is very similar to the results found by Schwaiger* et al.* [2]. It would be consistent with proton displacement controlling gating. It is less obvious why the physically larger tryptophan (in F233W) would lead to easier gating if the entire S4 had to move (Tao *et al.* [4] also compare gating with several synthetic amino acids; the interpretation of these results seems more complex, and we cannot compare them to our calculations, but they do not appear to contradict the conclusions we draw here.).

The rotation of the phenylalanine rings is large in the cysteine mutant; in WT, it may be difficult to rotate when the arginine is filling the space into which the rotation would move the side chain. However Schwaiger *et al*., still find that F233 has some room to rotate [2]. Taken together, these results support the idea that the accessibility of the cysteine is dependent on the conditions within the cavity left by the R→C mutation rather than the physical movement of the entire S4. The rotation of the F180 side chain alone appears able to cover and uncover the cys, making it possible for it to react, or blocking it; the effect of the F233 and the Y266 appears to be consistent with this. In addition, the state dependent rotation of the side chains probably also blocks/unblocks an entering MTS reagent. We can see in Table 1 that the distances of the phenylalanine rings (measured from the nearest hydrogen) to the cysteine sulfur change drastically as the protons move. Major changes in distance shown in Table 1 include the folding upward of F233 with one proton (it moves closer to R303, away from K306, as a single proton moves down (this can be seen in Figure 2) with the effect already present when the proton is at the mid position). F180 is closer to R297 with a single proton in the up position. With two protons (Table 2), F180 moves somewhat less, as there is a proton at the mid position (E226) that seems to be most important for this residue. F233 is about 3 Å closer to K306, and further from R303, with the protons middle and down (E226, K306), compared with middle and up (E183, E226). The rest of the distances show less change, but the phenylalanine distances make the rotations that are visible in the figures quantitative. The last column, in which the proton is on the cysteine itself, would correspond to a non-reactive regime, as an un-ionized cysteine does not react with MTS reagents [5]. We note that Schwaiger *et al*. [2], based on MD simulations, attributed the entire behavior of F233 to the van der Waals interactions with neighboring amino acids, the strongest being I230. However, MD simulations are limited not only by the classical potentials they must use, but, more importantly in this case, by the fact that they do not allow protons to transfer. A quantum calculation, even at HF level, includes not only dispersion interactions, but also the effects of polarization and charge transfer. Energy estimates presented here use a DFT calculation with large basis set (B3LYP/6-311G**), in a single point calculation. These should thus be quite accurate, and would include the effects of polarization and charge transfer. For the F233W mutant, one must include the strong effect of the larger ring current of the tryptophan π-electrons. Our results, like those of Schwaiger *et al*., also account for the effect of F233, although the major reason for the effect is not the same. Our calculation remains classical to the extent that it uses the Born-Oppenheimer approximation, thus does not allow for delocalization of the proton itself; whether such an effect matters cannot be determined from a straightforward extension of these calculations. It would be expected to affect hydrogen bonds shorter than 2.6 Å [44].

The energy change that accompanies the proton motion is given in Table 3.

At room temperature, 0.001 Ha ≈ k_B_T, so the 1 H^+^ down case is apparently at too high an energy to be relevant. Whether the same energy consideration applies to the WT channel would require a set of calculations on WT; these are left for future work. Because the single proton energy difference is very large going to the down state, we presumably must take two protons to have a realistic representation of the system (possibly a third proton should also have been included to provide adequate gating current, but we saw earlier that this may not be close to the main section of the VSD, hence would be of lesser importance.) If we look primarily at the two proton case, there is a difference of 0.006 Ha ≈ 6 k_B_T between the open configuration (H^+^ up + middle) and the closed (H^+^ down + middle), a very reasonable difference. The proton-field interaction energy would be larger than 0.006 Ha, approximately 0.004 Ha favoring the closed position, just as would be required.

**Table 3:** The total energy (*Hartrees: 1 Ha = 2625.5 kJ) of the systems in Figure 2 and Figure 3 (top to bottom in the Table correspond to (**A**–**C**) in the figures). These are single point energy calculations done using B3LYP/6-311G** using structures optimized with HF/6-31G**; 1 H^+^ case, 489 atoms (includes 6 water molecules), 2 H^+^ case, 493 atoms (includes 7 water molecules, accounting for the average difference in energy between the 1 H^+^ and 2 H^+^ cases).

Energy * for 1 and 2 H^+^	1 Proton	2 Protons
Up (1 H^+^) or (up + cys: 2 H^+^)	−12,354.5922	−12,431.4718
Middle (1 H^+^) or (up + middle: 2 H^+^)	−12,354.6023	−12,431.4760
Down (1 H^+^) or (middle + down: 2 H^+^)	−12,354.5670	−12,431.4698

MD simulations generally favor motion of the entire S4. Not all MD simulations lead to the same displacement. These are large and careful calculations, and have been recently reviewed [45], but as we have discussed proton transfer is not allowed: also, there are problems with water in nanoconfined spaces, different types of hydrogen bonds, polarization, variations in salt bridges with water content, and neglect of charge transfer. (It should be immediately acknowledged that there are problems with quantum calculations as well: the calculation is effectively at 0 K, and the energy minimum that is found is not always the global minimum. More important, distant sections of the system must be omitted. Therefore our calculation may also require some revision eventually). We can say, based on the calculations presented here, that the effects of R→C substitution are profound; it is not appropriate to assume that the channel is functionally unchanged by the mutation, with only a mechanical shift in S4 adequate to explain the experimental results.

There is evidence concerning gating other than MTS reactions with R→C mutated channels, which includes work on fluorescence transfer that has been interpreted in terms of the standard model [46,47], as well as a great deal of experimental evidence that could be more easily discussed in terms of alternative models (indeed, seem incompatible with standard models), such as effects of D_2_O substitution [48]. All this is reserved for separate consideration.

Taken together, it is clear that access measurements in the R300C mutant would be very different if protons are near the extracellular end, near the intracellular end, or in the middle, even if there were no motion of the backbone of the S4 segment at all. This by itself does not conclusively prove that the proton positions are the cause of the channel being open and closed, nor even that they are correlated with the open and closed conformations. However, the results found here are just what would be expected if they are the cause. In addition, these results suggest strongly that interpreting the access of the cysteine to MTS reagents as being proof, or even indicative of, motion of the cysteine to the membrane surface, is at least premature. Cysteine is so different from arginine that it is not possible to consider the MTS reactions in the mutated channel as being definitive evidence for the position of arginine in the open or closed state of the WT channel.

It would be necessary to extend these calculations to all the R→C mutations that have been done on this, and similar, channels, and repeat for several specific MTS reagents, to have conclusive evidence that these mutations fail to prove S4 motion. However even this single mutation shows how the standard interpretation needs to be reexamined; because the computer resources needed to extend this work are so substantial, we offer as much as is presently possible, to show the key points that need consideration.

## 3. Conclusions

(1) An R300C mutation creates a large cavity in the VSD. This allows three aromatic residues: two phenylalanines (F233, near the intracellular side of the VSD, (down), and F180 from S1, nearer the extracellular side (up)), and a tyrosine, Y266, just below the C300, to swing their aromatic rings, in some cases through angles of over 100°, blocking different parts of the channel; Y266 forms a hydrogen bond to the sulfur of C300.

(2) The blocked sections can include the cysteine that has been inserted in an R300C mutation.

(3) The blocked section, and the direction (extracellular/intracellular) from which it is blocked, depends on the position of water and protons; this appears to correlate with the open/closed state of the channel. Although further work would be needed to confirm causality, this is a strong indication that proton motion could produce the experimental results with the R300C mutation.

(4) The (extracellular/intracellular) position of the cysteine residue cannot be inferred from the results of reaction with MTS reagents made available from either the intracellular or extracellular side. The position of the cysteine itself does not change appreciably with the protons and water, unlike the side chains of the aromatic amino acids, although the carbon-sulfur bond does rotate slightly. This effect is small; see Table 1 and Table 2. Cysteine reactivity appears to have more to do with the proton and water position effects on the aromatic rings of F180, F233 and Y266. With the protons up (channel open, in this model), there appears to be space above the cysteine for a MTS reagent; below seems less conclusive, but it does appear that access would be blocked from below. With the proton in any position, Y266 makes a hydrogen bond to the cysteine. The Y266 side chain oxygen to cys S distance changes little (see Table 1 and Table 2), and is always close enough for the H of the Y266 –OH to hydrogen bond to the cys S. With a proton only in the middle, the F180 folds out of the way, so reaction with an extracellular MTS is possible, but not with an intracellular MTS; a nearby hydrogen bond should affect reactivity. F233 also rotates, blocking access from the intracellular side for some proton positions. Addition of a third H^+^ may make some difference, but if the aromatic residues follow the H^+^ this effect may be additive but not qualitatively different.

(5) Conversely, with the proton or protons down, the intracellular path to the cysteine appears open, but the F180 blocks the reagent above.

(6) If, as we have earlier proposed, protons are responsible for the gating current, they would also be responsible for the differential access to the cysteine sulfur. The calculation shows that this is plausible.
